# Annotations

**Published:** 1906-10-20

**Authors:** 


### ANNOTATIONS.

### The Late Dr. James Finlayson.

The medical profession in general, and the Glasgow School in particular, have suffered a severe loss by the death of Dr. Jas Finlayson. Apparently in his usual health, and actively engaged in his professional duties, he was suddenly attacked with apoplexy on the 9th inst. and died in the course of a few hours. He will probably be best known to the profesion generally by his "Clinical Manual" and by his numerous writings on the diseases of children and on the history of medicine. But in his own school and city these claims to respect were reinforced by a long record of efficient service as a consulting and hospital physician and clinical teacher, and by the memory of many other duties discharged quietly and unobtrusively, and ever with true and unselfish motive. Still further, Dr. Finlayson created personal associations which, among all who knew him, will cause his memory to be cherished with deep and sincere regard. Of the many hundreds of students who have passed through his classes, none can fail to have been favourably directed by the sustained attention and thoroughness with which all cases of disease entering the wards were investigated, and by the gentleness and unaffected kindliness extended to every patient. As a consultant Dr. Finlayson commanded general confidence among both the profession and the public, and for many years he has occupied a leading position in the West of Scotland. For more than a quarter of a century he acted as Honorary Librarian to the Faculty of Physicians and Surgeons, and to him largely the present value of the library is due. From 1900 to 1903 he was President of the Faculty, which is under deep obligations to him for his many services admirably performed; not the least of these is his "Life of Maister Peter Lowe," and his bibliographical demonstrations of ancient medical classics. In 1899 he received the honorary degree of LL.D. from his Alma Mater—an honour well deserved. Of a quiet and gentle disposition, and animated by a high sense of duty and responsibility, Dr. Finlayson represented the best traditions of the profession. He served medicine loyally and with none but worthy ambitions.

### The Pauper's Pipe.

The conundrums which are brought before Guardians to solve are often of rather a humorous nature. Among these may be set the request which was recently brought before the Pontefract Board by an old woman who asked that she might be allowed tobacco as she was "accustomed to the weed." Old men, as well as those who do any special work, are generally allowed a certain amount of tobacco weekly, but the old ladies, as they always call themselves, generally have their indulgence granted in the form of tea. It is doubtful if since the memorable, if not historic, occasion when Oliver Twist asked for a second supply of gruel, such a bewildering request was ever preferred to a Poor-law authority. Though doubtless many of the old crones who go to the workhouse to end their days have indulged in an occasional pipe, it is probable that this is the first that expected the indulgence to be continued there. The Pontefract Vice-Chairman, probably speaking humorously, advocated the limitation of female smoking to cigarettes, but we fear that a cigarette, or even two, would give but small satisfaction to a lady accustomed to a clay pipe filled with good strong twist. Ultimately the chairman moved that "the practice of smoking be allowed only under medical direction," and, falling in with this view, the Board, by a majority of votes, consented to the applicant being allowed to smoke, but only on the doctor's recommendation. One cannot but hope that the doctor will himself be enough of a smoker to have a fellow-feeling for the old woman. If not, we hope that some one will bring to his mind Mark Twain's saying about the dangers attached to giving up familiar habits—especially bad ones.

### Dental Philanthropy.

Mr. George Cadbury, not content with what he has already done in the way of philanthropy, has taken up a new plan for promoting the health of the people. He has offered to provide about five thousand school children in the King's Norton district with tooth-brushes and tooth-powder. He hopes in this way to do something towards preventing physical deterioration. To old-fashioned folks this may seem rather a comical offer, and any one who knows how difficult it is to make children brush their teeth as often as they should, will fear that to some extent the effort may fail. But if even a part of the children use the brushes and powder no little good will be done. It is sad to see among our poorer classes quite young and comely men and women disfigured by the absence of teeth or by their presence only in the form of decayed stumps, almost useless, and liable to cause toothache and indigestion. Among the lower classes the use of the tooth-brush is almost unknown, and the dentist is recognised only as the extractor and by no means as the preserver of teeth. We know that many otherwise eligible recruits for the army have to be rejected on account of the state of their teeth, and we know also that serious illnesses arise from the swallowing of imperfectly masticated food, due to the fact that the teeth are unfit to do their proper work. We have much to learn from the Americans in the care of the teeth. They believe in the superiority of prevention, and a periodical visit to the dentist is a part of the year's routine. The perfection of their dentistry is shown by the fact that many who have no claim to the description call themselves "American dentists" in order to attract clients. The Americans know how to preserve every possible piece of their patients' own teeth as well as to replace them when necessary. But the real American dentists have wiser people for patients than those here; people who know that in cleanliness lies the source of comfort and well-being for the teeth. The use of the tooth-brush goes farther down the social scale than it does here, and not even the humblest would look upon it as a mere article of luxury. If Mr. Cadbury's gift helps to bring about a similar state of affairs here, he will indeed have done something to improve public health and prevent physical deterioration.

